# Atraumatic Splenic Rupture: A Case Report

**DOI:** 10.7759/cureus.102979

**Published:** 2026-02-04

**Authors:** Andreia Salgadinho Machado, Miguel Silvestre, Marta Roldão, Marta Anastácio, Ana Margarida Ribeiro

**Affiliations:** 1 Internal Medicine, Hospital São Francisco Xavier, Lisbon, PRT; 2 General Surgery, Hospital São Francisco Xavier, Lisbon, PRT

**Keywords:** acute abdomen, atraumatic splenic rupture, diffuse large b-cell lymphoma, hematologic malignancy, splenectomy

## Abstract

Atraumatic splenic rupture (ASR) is a rare but potentially fatal emergency, accounting for less than 0.5% of all splenic ruptures. Its nonspecific presentation often complicates diagnosis and management. True idiopathic ASR, occurring without an underlying splenic disease, is extremely rare.

We report the case of a 61-year-old woman with diffuse large B-cell lymphoma in complete metabolic remission who developed sudden, severe epigastric and left upper quadrant pain 7 days after receiving high-dose methotrexate for central nervous system prophylaxis. There was no evidence of splenic involvement by lymphoma or other predisposing factors. The patient rapidly progressed to hemorrhagic shock. Imaging identified a large subcapsular splenic hematoma with active bleeding, necessitating emergent splenectomy. Following surgery, she achieved hemodynamic stabilization and recovered without postoperative complications, remaining disease-free at the six-month follow-up. Histopathological examination confirmed preserved splenic architecture without malignancy, vascular pathology, or amyloid deposition.

This case highlights the risk of diagnostic anchoring bias in patients with a history of hematologic malignancy, as ASR may be incorrectly attributed to disease recurrence. In the absence of splenic involvement or identifiable risk factors, histopathological confirmation was essential to establish the diagnosis of true idiopathic ASR and to guide appropriate management.

## Introduction

Atraumatic splenic rupture (ASR) is a rare, potentially fatal emergency, representing less than 0.5% of all splenic ruptures [1]. Unlike traumatic splenic rupture, ASR occurs without a history of trauma and often presents with nonspecific symptoms, leading to frequent misdiagnosis or delay [1,2]. ASR is classified as atraumatic-pathological, associated with underlying splenic disease, or atraumatic-idiopathic, occurring in a histologically normal spleen without identifiable triggers [1].

A large systematic review found that about 7% of ASR cases are idiopathic, while 93% are linked to underlying conditions [1,2], most commonly hematological malignancies (such as leukemia and lymphoma), infections (including infectious mononucleosis and pancreatitis), inflammatory diseases (such as amyloidosis or vascular disorders), and drug-related factors, especially anticoagulant use [1,3].

The hallmark of ASR is sudden left upper quadrant pain, which may radiate to the left shoulder (Kehr’s sign). This finding occurs in 20% to 50% of cases [1,4].

Management depends on hemodynamic stability and the underlying cause. Non-operative approaches, such as close monitoring or splenic artery embolization, may be suitable for stable patients [5,6]. Emergency splenectomy remains the preferred treatment for ongoing hemorrhage or clinical instability [1,5].

We present a case of ASR during high-dose methotrexate (MTX) therapy in a patient with diffuse large B-cell lymphoma (DLBCL) in complete remission, without splenic involvement. This case highlights the diagnostic challenges of ASR and the need for rapid imaging and immediate surgical intervention in unstable patients.

## Case presentation

A 61-year-old woman with a history of active smoking was diagnosed in July 2022 with diffuse large B-cell lymphoma (DLBCL), non-Hodgkin B-cell type, stage IE, involving the nasopharynx. The hematology team recommended six cycles of Rituximab, Cyclophosphamide, Doxorubicin, Vincristine, and Prednisone (R-CHOP), followed by two cycles of high-dose methotrexate (MTX) for central nervous system prophylaxis.

One month after completing R-CHOP, positron emission tomography showed a complete metabolic response. Laboratory evaluation revealed preserved bone marrow, renal, and hepatic function (Table 1). No additional supportive therapies, including granulocyte colony-stimulating factor, were required.

**Table 1:** Evolution of laboratory parameters one week before and in the days following methotrexate administration 1 week before MTX: outpatient setting; 1 day before MTX, 3 days after MTX, and 7 days after MTX: inpatient setting MTX: methotrexate; AST: aspartate aminotransferase; GOT: glutamic oxaloacetic transaminase; ALT: alanine transaminase; GPT: glutamate-pyruvate transaminase; GGT: gamma-glutamyl transferase; LDH: lactate dehydrogenase; CK: creatine kinase; aPTT: activated partial thromboplastin time; INR: international normalized ratio

Parameter	Reference range	1 week before MTX	1 day before MTX	3 days after MTX	7 days after MTX
Red Blood Cells (x10¹²/L)	3.85 – 5.00	4.3	3.61	3.25	3.04
Hemoglobin (g/dL)	12.0 – 15.0	12.1	10.8	9.7	9.1
Hematocrit	0.360 – 0.460	0.369	0.314	0.283	0.267
White Blood Cells (x10⁹/L)	4.0 – 10.0	6.1	4.3	2.4	3.0
Platelets (x10⁹/L)	150 – 400	272	194	145	199
Urea (mg/dL)	17 – 49	28	20	12	47
Creatinine (mg/dL)	0.50 – 0.90	0.6	0.52	0.41	0.50
Total Bilirubin (mg/dL)	<0.15	<0.15	<0.15	0.22	0.19
AST / GOT (U/L)	<32	17	17	41	35
ALT / GPT (U/L)	<33	19	21	49	75
Alkaline Phosphatase (U/L)	35 – 104	73	62	-	66
GGT (U/L)	6 – 42	9	9	-	12
LDH (U/L)	135 – 225	159	161	211	166
CK (U/L)	22 – 200	-	22	-	20
Troponin T (ng/L)	<5	-	15	-	20
Myoglobin (µg/L)	<21	-	<21	-	< 21
Lipase (U/L)	0 – 160	-	17	-	18
Sodium (mmol/L)	136 – 145	145	147	145	139
Potassium (mmol/L)	3.50 – 5.10	4.27	3.98	3,5	4.46
Calcium (mg/dL)	8.8 – 10.2	-	8.4	8.5	8.5
C-Reactive Protein (mg/dL)	<0.50	0.32	0.56	1.54	6.2
aPTT (s)	25 – 35 s	-	29	-	30
Prothrombin Time (s)	11 – 14 s	-	12.2	-	12.5
INR	0.8 – 1.2	-	1.0	-	1.1

One week later, the patient was admitted for her first MTX cycle. Three days after infusion, she developed a transient fever without focal symptoms, which resolved with antipyretics. She remained hemodynamically stable, with normal oxygen saturation and no splenomegaly. Laboratory tests showed pancytopenia and MTX levels below 0.04 µmol/L. No further investigation of pancytopenia was performed, including tests for parvovirus B19, Cytomegalovirus, Epstein-Barr virus, vitamin B12 or folate deficiency, or coagulation abnormalities.

Seven days after MTX administration, the patient developed sudden severe epigastric pain radiating to the left upper quadrant and chest, accompanied by nausea, diaphoresis, and presyncope. She denied recent trauma or significant physical exertion. On examination, she was tachycardic and mildly hypotensive, with abdominal tenderness and guarding. Despite initial fluid resuscitation, she rapidly progressed to refractory hemodynamic instability requiring vasopressor support. Laboratory evaluation showed acute anemia with a preserved platelet count and normal coagulation parameters, without elevations in pancreatic or cardiac enzymes (Table 1). The initial arterial blood gas analysis showed no significant abnormalities (Table 2).

**Table 2:** Evolution of arterial blood gas parameters during acute clinical deterioration

Parameter	Reference Values	Initial assessment	2 hours later
pH	7.350 – 7.450	7.395	7.347
pCO₂ (mmHg)	35.0 – 45.0	40.2	37.3
pO₂ (mmHg)	75.0 – 100.0	62.8	136
HCO₃⁻ (mmol/L)	21 – 28	24.1	19.9
Anion Gap (mmol/L)	7 – 16	6.6	16.6
Hematocrit (%)	36-44	28	16
Hemoglobin (g/dL)	12.0-16.0	9.1	5.0
Sodium (mmol/L)	135 – 148	138	140
Potassium (mmol/L)	3.50 – 5.00	3.6	3.9
Ionized Calcium (mg/dL)	4.5 – 5.3	4.6	4.6
Chloride (mmol/L)	99 – 106	111	108
Lactate (mmol/L)	0.50 – 2.00	1.8	3.6

**Figure 1:**
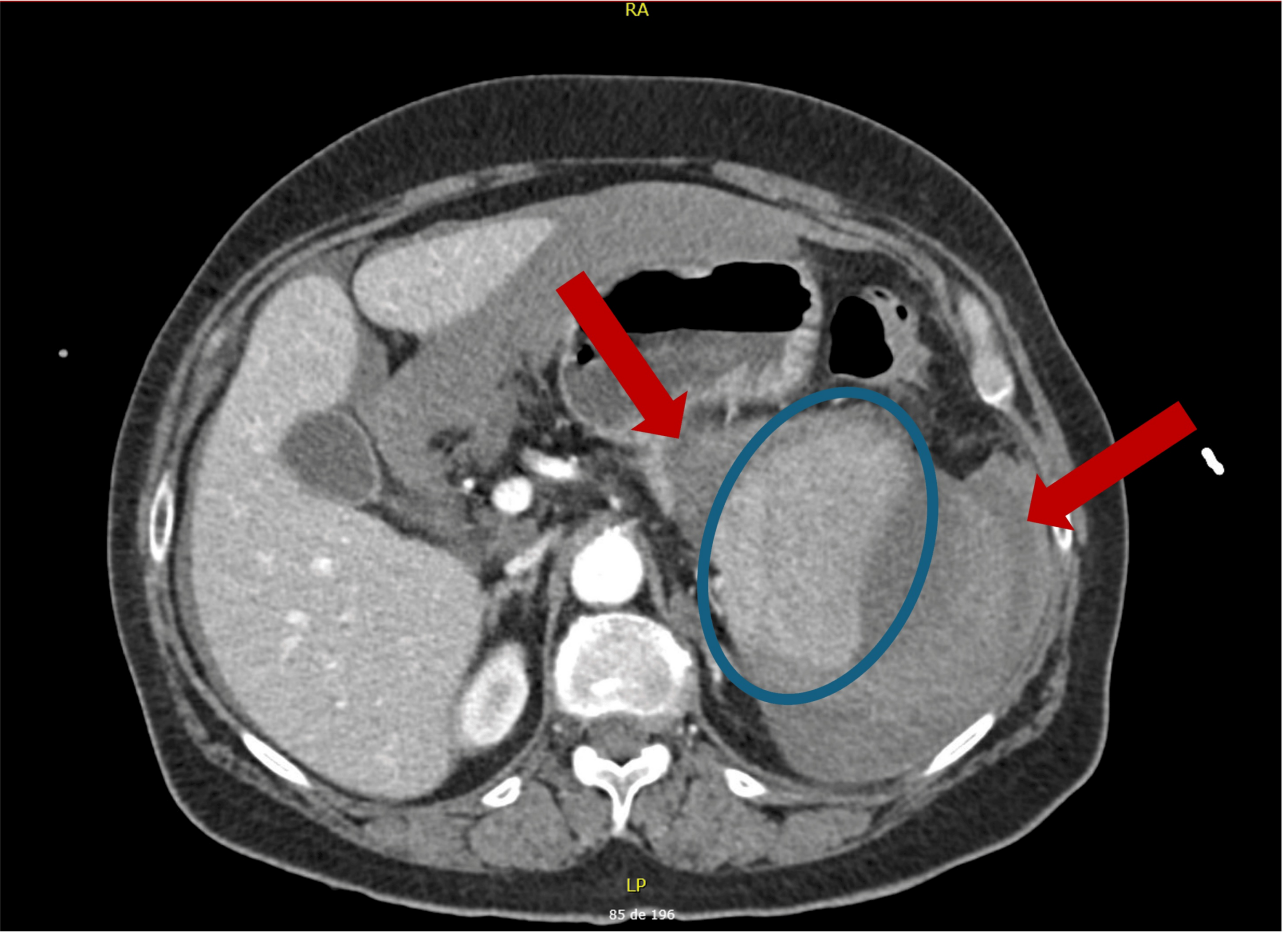
Axial contrast-enhanced CT of the abdomen and pelvis blue circle: large subcapsular hematoma; red arrows: active contrast extravasation

**Figure 2:**
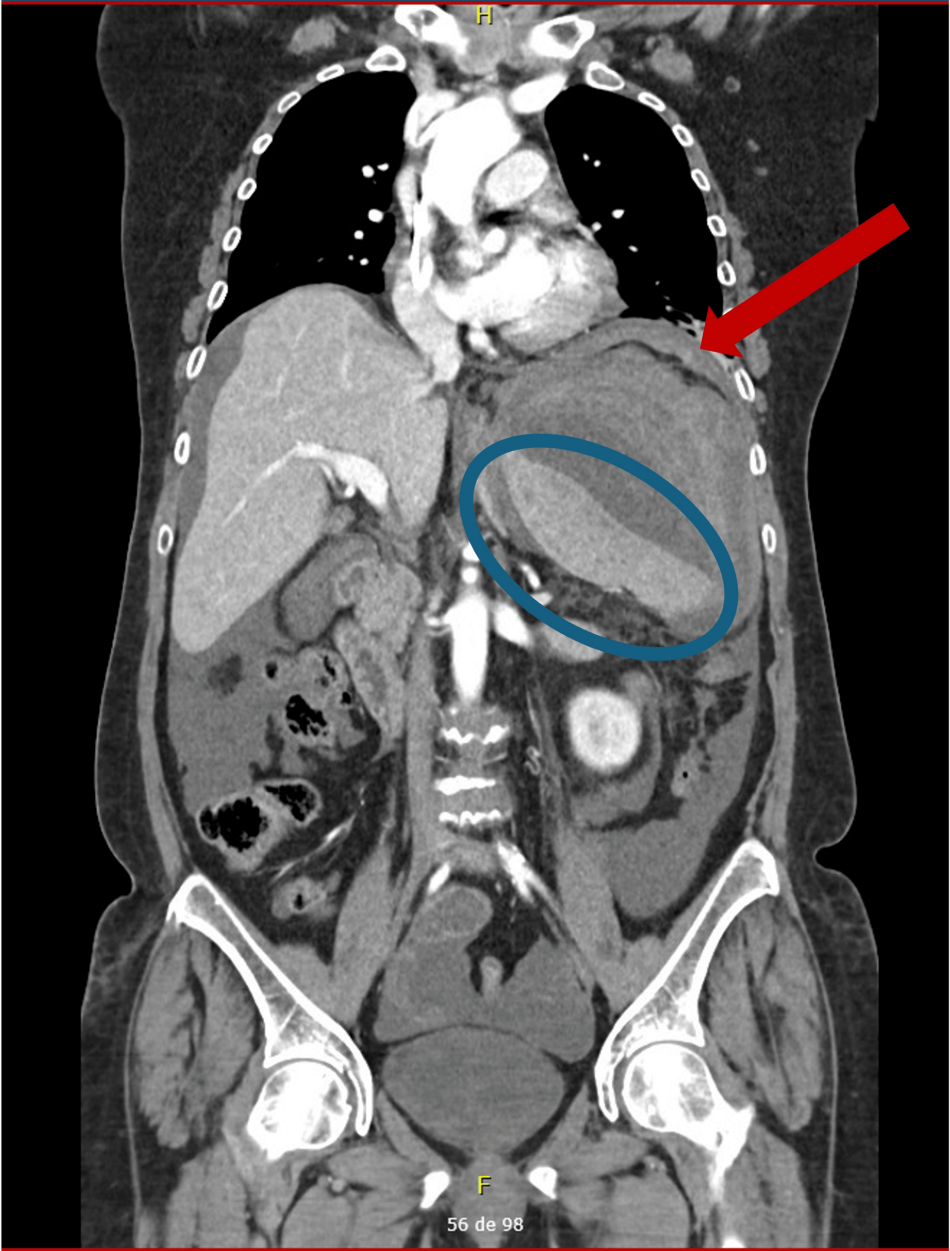
Coronal contrast-enhanced CT of the abdomen and pelvis blue circle: large subcapsular hematoma; red arrows: active contrast extravasation

Electrocardiography and chest and abdominal radiography were unremarkable. An extended focused assessment with sonography for trauma (eFAST) identified left perirenal free fluid. Abdominal and pelvic CT scan showed a large subcapsular splenic hematoma (16 × 12 × 14 cm) with active contrast extravasation, compression of a homogeneously enhancing spleen, and moderate hemoperitoneum, consistent with ASR (Figures 1, 2).

Two hours later, repeat arterial blood gas analysis demonstrated a rapid decline in hemoglobin to 5 g/dL, accompanied by a marked rise in serum lactate to 3.6 mmol/L, consistent with ongoing hemorrhagic shock (Table 2). A massive transfusion protocol was initiated, including three units of packed red blood cells, three units of fresh frozen plasma, and one platelet pool. The patient underwent emergent splenectomy, with an estimated intraoperative blood loss of 2,200 mL. Postoperatively, additional red blood cell and platelet transfusions were administered, guided by rotational thromboelastometry. She was admitted to the intensive care unit for 24 hours before transfer to the hematology ward.

Histopathological examination of the spleen revealed a large subcapsular hematoma without evidence of lymphomatous infiltration, vascular pathology, or amyloid deposition, confirming the atraumatic nature of the rupture. The postoperative course was uneventful, and the patient was discharged one week later. At the six-month follow-up, she remained clinically well with no evidence of disease relapse or recurrence.

## Discussion

ASR, though rare, presents significant diagnostic and therapeutic challenges and carries a high mortality risk if not promptly recognized [1,3]. The lack of preceding trauma and nonspecific presentation often leads to misdiagnosis or delay, requiring a high index of suspicion in patients with unexplained acute abdominal pain or shock [1,2]. Typical signs include sudden left upper quadrant pain, abdominal guarding, and hemodynamic instability.

In this case, despite a history of lymphoma, histopathological examination revealed preserved splenic architecture without lymphomatous infiltration, vascular abnormalities, or amyloid deposition, fulfilling criteria for true idiopathic ASR. Although the patient had recently completed intensive chemotherapy, she was in complete metabolic remission and had not received granulocyte colony-stimulating factor, which is a known trigger of splenic engorgement [6]. Currently, there is no evidence supporting an association between methotrexate and ASR [7]. However, the cumulative toxicity of six cycles of R-CHOP may have contributed to subclinical alterations in splenic parenchyma, as chemotherapy has been shown to induce microscopic structural and vascular changes [4].

Contrast-enhanced CT remains the gold standard for diagnosing ASR, allowing accurate detection of hemoperitoneum, assessment of splenic injury, and identification of active bleeding. While focused abdominal ultrasound may be useful as an initial screening tool in unstable patients, it can miss low-grade injuries or small fluid collections [2,5].

Management is primarily determined by hemodynamic stability. In hemodynamically stable patients without active contrast extravasation, conservative approaches, such as close monitoring, intravenous fluids, analgesia, and repeat imaging, may be considered. Unstable patients require emergent splenectomy, which provides definitive hemorrhage control and allows for histopathological confirmation, which is essential to distinguish between malignancy-related rupture and idiopathic events in patients with hematologic disease [1,5]. In this case, rapid clinical deterioration with refractory shock and a significant drop in hemoglobin necessitated immediate surgery, which proved life-saving.

Although overall ASR mortality has decreased to 8.7% with advances in imaging and resuscitation, the risk remains significant, especially in patients over 50 [1]. In this case, prompt detection and immediate surgery led to complete recovery.

Postoperative management requires lifelong prophylaxis, including vaccinations against encapsulated organisms such as *Streptococcus pneumoniae*, *Haemophilus influenzae*, and *Neisseria meningitidis *to prevent overwhelming post-splenectomy infection [4,8]. After discharge and vaccination, the patient remained under outpatient general surgery care for six months and was alive at the last follow-up.

Clinicians should consider ASR in patients with acute abdominal pain or unexplained shock, even without a history of trauma or known splenic disease. Early recognition, prompt imaging, and timely surgery are critical to improving outcomes.

## Conclusions

ASR is a rare, potentially fatal condition that requires prompt recognition and intervention. This case is notable for occurring in a patient with DLBCL in complete remission, without histopathological evidence of splenic involvement. The absence of lymphomatous infiltration, vascular pathology, or amyloid deposition allowed classification as true idiopathic ASR. In patients with a history of lymphoma, there is a risk of diagnostic anchoring bias, where splenic rupture is presumed to be malignancy-related. This case underscores the importance of avoiding such assumptions, as delayed recognition can lead to catastrophic outcomes. Early consideration of ASR, rapid contrast-enhanced CT imaging, and prompt surgical intervention were critical to achieving a favorable outcome.

Continued reporting of idiopathic ASR cases in patients with hematologic malignancies in remission is essential to improve the understanding of its pathophysiology, refine diagnostic strategies, and reduce delays in similar scenarios.
